# White Mulberry (*Morus alba*) Foliage Methanolic Extract Can Alleviate *Aeromonas hydrophila* Infection in African Catfish (*Clarias gariepinus*)

**DOI:** 10.1155/2014/592709

**Published:** 2014-12-10

**Authors:** Atefeh Sheikhlar, Abd Razk Alimon, Hassan Daud, Chee R. Saad, Carl D. Webster, Goh Yong Meng, Mahdi Ebrahimi

**Affiliations:** ^1^Department of Animal Science, Faculty of Agriculture, Universiti Putra Malaysia, 43400 Serdang, Selangor, Malaysia; ^2^Aquatic Animal Health Unit, Department of Veterinary Clinical Studies, Faculty of Veterinary Medicine, Universiti Putra Malaysia, 43400 Serdang, Selangor, Malaysia; ^3^Department of Aquaculture, Faculty of Agriculture, Universiti Putra Malaysia, 43400 Serdang, Selangor, Malaysia; ^4^Aquaculture Research Center, Kentucky State University, Frankfort, KY 40601, USA; ^5^Department of Veterinary Preclinical Sciences, Faculty of Veterinary Medicine, Universiti Putra Malaysia, 43400 Serdang, Selangor, Malaysia; ^6^Institute of Tropical Agriculture, Universiti Putra Malaysia, UPM, 43400 Serdang, Selangor, Malaysia

## Abstract

Two experiments were simultaneously conducted with *Morus alba* (white mulberry) foliage extract (MFE) as a growth promoter and treatment of *Aeromonas hydrophila* infection in separate 60 and 30 days trail (Experiments 1 and 2, resp.) in African catfish (*Clarias gariepinus*). In Experiment 1, four diets, control and control supplemented with 2, 5, or 7 g MFE/kg dry matter (DM) of diet, were used. In Experiment 2, fish were intraperitoneally infected with *Aeromonas hydrophila* and fed the same diets as experiment 1 plus additional two diets with or without antibiotic. Results of experiment 1 showed that growth was unaffected by dietary levels of MFE. Treatments with the inclusion of MFE at the levels of 5 and 7 g/Kg DM had no mortality. Red blood cells (RBC), albumin, and total protein were all higher for the treatments fed MFE (5 and 7 g/Kg DM). Results of experiment 2 showed RBC, hemoglobin, hematocrit, globulin, albumin, and total protein improved with the increase in MFE in the infected fish. The dietary MFE at the level of 7 g/kg DM reduced mortality rate. In conclusion, MFE at the level of 7 g/kg DM could be a valuable dietary supplement to cure the infected fish.

## 1. Introduction

Antibiotics have been extensively used as growth enhancer and immunity enhancer and treatment of bacterial diseases in fish. However, the application of antibiotics and other chemotherapeutics has negative aspects such as risk of creating resistant pathogens, problems of antibiotic residues accumulating in treated fish, and unfavorable impact on the environment [1, 2]. Hence, the European Union has banned the application of antibiotics and other chemicals that were efficient in promoting animal growth (Regulation 1831/2003/EC); therefore, the demand for replacement of natural products has been rising with a focus on plant products as alternative to antibiotics. Various herbs and their extracts have been used experimentally for this purpose in several studies [3–6]. Bansemir et al. [3] showed that the oregano essential oil improved disease resistance to pathogens when supplemented to channel catfish feed. Sivaram et al. [7] showed that* Ocimum sanctum* (OS) and* Withania somnifera* (WS) can act in juvenile greasy grouper fish as pathogen inhibitor.

*Morus alba*, commonly known as white mulberry, is a tree which is native of India, China, and Japan. In the past, mulberry was mainly cultivated for the silkworm industry. However, researches over the last two decades have shown the benefits of it as ruminant and monogastric feeds [8–11]. Furthermore,* Morus alba* has a long history of therapeutic application in Chinese medicine [12] and the foliage, in particular, showed antimicrobial [13], antifungal [14], antiallergic [15], antioxidant [16], and antihypoglycemic [17] activities. White mulberry leaf contains secondary metabolites including tannins, phytosterols, sitosterols, saponins, triterpenes, flavanoids, benzofuran derivatives, morusimic acid, anthocyanins, anthroquinones, glycosides, and oleanolic acid [18–20]. There is, however, no information on the therapeutic effect of* Morus alba* extract in catfish. Therefore, it was hypothesized that the methanolic extract of* Morus alba* foliage (MFE) might provide protection against microbial infection in fish and might also act as the dietary fish growth promoter. The hypothesis was tested in the present two experiments with African catfish (*Clarias gariepinus*) fingerlings and the results are discussed in the present study. The possible use of active ingredients of* Morus alba* foliage for prevention or treatment of the bacterial fish diseases should be discussed.

## 2. Materials and Methods

### 2.1. Preparation of Herbal Extract

Fresh* Morus alba* foliage (MF) was harvested and then air-dried. The dried MF was powdered using electric blender. A sample (100 g) of the powder was added into a conical flask containing 1000 mL of 95% methanol; the flask was then kept for seven days at room temperature and agitated every day. Afterward, the supernatant was removed and the methanol content was evaporated by a rotary evaporator; the residue was freeze-dried (Labconco, USA) and stored in a sterile bottle at −20°C until used [21].

### 2.2. Diets and Treatments

In the Experiment 1 a control diet (CNT) was formulated (Table 1) according to the NRC [22] recommendation for fish which contained 35% crude protein and 3498 kcal of digestible energy/kg of dietary dry matter (DM). Additional three diets were the control diet supplemented with 2, 5, or 7 g MFE/kg DM (MFE-2, MFE-5, or MFE-7, resp.), prepared according to Zilberg et al. [23]. All dry ingredients were ground and mixed using a food mixer. Distilled deionized water was added into each diet (1.2 mL/mg DM) and mixed. The mixed diets were pelleted (2 mm diameter), air-dried (at 23 C for 72 h with air condition and electrical fan), and kept frozen (−20°C) in airtight plastic bags. In the Experiment 2 the fish received the same diets as in Experiment 1. However, in the Experiment 2, the control diet was CNT2 and two additional dietary treatments were added to the experimental design, namely, CNT2-I and CNT2-I-A. Consequently, in the Experiment 2 the treatment CNT2-I was fish infected (I) with* A. hydrophila* and fed the control diet without any additive, while the treatment CNT2-I-A was fish infected with* A. hydrophila *and fed the control diet supplemented with the antibiotic (A) oxytetracycline (1 g 199/g of dietary DM). Therefore, there were 6 treatments (CNT2, CNT2-I, CNT2-I-A, MFE-2-I, MFE-5-I, and MFE-7-I) in the Experiment 2. On day 1, all fish in each aquarium with treatments CNT2-I, CNT2-I-A, MFE-2-I, MFE-5-I, and MFE-7-I were anesthetized with 50 mg/L of tricaine methanesulfonate (MS-222; Argent Chemical Laboratories, Redmond, WA) and infected with* A. hydrophila* using intraperitoneal injection of bacterial suspension (50 *μ*L/fish) containing 2.88 × 10^6^ cfu/mL of normal saline (according to LD_50–96 h_ determination). The fish kept to feed the assigned diets for 30 d.

### 2.3. Fish and Rearing System

Fingerling African catfish (*Clarias gariepinus*) were obtained from a local farm and fed a commercial diet for two weeks. Thereafter the fish were weighed and kept in 100 L aquaria connected to a recirculation system with 30 fish per aquarium. The initial weight (mean ± SE; *n* = 30) per fish was 8.4 ± 0.2 g. Each experimental diet was fed to three replicates of fish in two experiments. The first and second experiment lasted 60 and 30 days, respectively. There was a nonstop aeration to maintain the dissolved oxygen at the point of saturation. All fish were hand-fed two times daily at 4% of body weight and the daily ration was adjusted accordingly.

### 2.4. Water Quality (Experiments 1 and 2)

The aquaria were washed and cleaned weekly, the water quality (dissolved oxygen, temperature, ammonia, and pH) was checked daily; the dissolved oxygen and temperature were determined by YSI oxygen meter and thermometer, respectively. The ammonia-N was determined daily using a Fresh Water Aquaculture Kit Model AG-2 (LaMotte, Chestertown, Maryland, USA), while pH was measured by a pH meter (Fisher Scientific, Denver, USA). The photoperiod was 12 L: 12 D according to Buentello et al. [24]. The temperature was kept between 28 and 30°C, pH at 6.00 ± 0.03, and ammonia-N concentration was always lower than 0.05 mg/L. The photoperiod was 12 L: 12 D.

### 2.5. Weighing and Sampling

Fish in each aquarium were group weighed following a 24 h fasting. At the termination of each experiment, blood sampling was performed 24 h after the last feeding following anesthesia with 50 mg/L of tricaine methanesulfonate (MS-222; Argent Chemical Laboratories, Redmond, WA). Blood samples (heparinized and nonheparinized) were collected from the caudal vein of six fish randomly selected from each replicate for determination of red blood cells (RBC), white blood cells (WBC), and hematocrit (Ht) as reported previously [25–28]. Hemoglobin (Hb) was determined colorimetrically by measuring the formation of cyanomethemoglobin according to the method of van Kampen and Zijlstra [29].

The serum from nonheparinized samples were used for determination of total protein (TP) and activities of alanine amino transferase (ALT), aspartate amino transferase (AST), and alkaline phosphatase (ALP) using commercial kits and an autoanalyzer (Technicon RA-1000). Total lipids and albumin were determined colorimetrically according to the methods of Knight et al. [30] and Wotton and Freeman [31], respectively. Globulin was determined by the subtraction of albumin from total protein. The collected fish were euthanized in a solution of 200 mg/L of MS-222 and livers were removed for measurement of hepatosomatic index.

Samples of diets were analyzed for DM, crude protein, crude fat and ash according to AOAC [32]. The Kjeldahl method was used for determination of crude protein (N × 6.25). Crude lipid was measured by chloroform-methanol extraction [33]. Crude fiber was quantified according to loss of residue on burning at 600°C following hydrolysis of a sample in H_2_SO_4_ and NaOH. Gross energy was determined by bomb calorimeter (IKA-calorimeter C7000 Janke & Kunkel IKA Analysentechnik, Staufen, Germany) using benzoic acid as a standard. All diets were analyzed in duplicate. Phytochemical screening tests were conducted to determine the presence of phytocompounds in the extract. The phytocompounds include volatile oils, terpenoids, phenols, tannins, flavonoids, alkaloids, saponins, and steroids, which were determined based on the methods reported by Trease and Evans [34]; Sofowora [35]; Evans [36]; Houghton and Raman, [37]; Harborne [38]; Dahiru et al. [39]; Aiyegoro and Okoh [40].

### 2.6. Calculations

The following equations were used for calculation of results.

Feed conversion ratio (FCR) = feed DM intake (g)/(final biomass (g) − initial biomass (g) + biomass of the dead fish (g)).

Specific growth ratio (SGR) = 100 × ln (final weight/initial weight)/days of the experiment.

Hepatosomatic index (%) = (liver weight (g)/body weight (g)) × 100.

### 2.7. Bacterium

The bacterium used in the present experiment was* Aeromonas hydrophila *wasisolated from diseased fish and identified using the API 20E (Bio-Merieux, L'Etoile, France). It was cultured in brain, heart infusion (BHI) media (broth and agar) for 24 h at 37°C. The stock culture was stored at −80°C in 0.85% NaCl with 20% glycerol (v/v) in BHI broth to keep a stable inoculate during each experimental period.

### 2.8. Liver and Kidney Analysis

Over a 30 d postinfection period, mortality, appearance, appetite, and behavior of fish were recorded. Dead fish were removed daily; the liver and kidney of 10% of dead fish were removed aseptically and subjected to bacterial determination [41]. Bacteria isolated from dead fish were identified by the API 20E (Bio-Merieux, L'Etoile, France). All the surviving fish on day 30 were euthanized in a solution of 200 mg/L of MS-222 and the kidney and liver were removed for determination of the presence or absence of A. hydrophila.

### 2.9. Median Lethal Dose (LD_50_) of* Aeromonas hydrophila*

Nine treatment groups with three replicates, each of ten healthy fish with an average weight of 9 g ± 0.3, were used to determine the pathogenicity (LD_50_) of* A. hydrophila* [42]. The fish were fed twice daily a commercial diet to* ad libitum* feed intake. After five days of acclimatization period the fish in eight treatment groups were injected intraperitoneally with 50 *μ*L of bacterial suspension containing graded concentrations of bacterium, ranging from 10^8^ to 10^1^ CFU/mL. The ninth group was injected with 50 *μ*L of 0.9% normal saline and served as a control group. The experiment lasted 96 h and dead fish were removed from the aquarium daily. The liver and kidney of the dead fish were used for identification of* A. hydrophila*. The LD_50_ was determined for 96 h as log LD_50_ = *α* log* b*  + * c*, where *α*  = [mortality > 50% − 50%]/[mortality > 50% − mortality < 50%],* b*  = dilution rate (10^−1^) and* c*  = the log of the minimum dilution rate in which the mortality was 50%. The LD_50_ was expressed as CFU/mL.

### 2.10. Statistical Analysis

All the data were analyzed by the GLM procedure of SAS software. Duncan's multiple range tests were used to determine significance of differences among treatment means at *P* < 0.05. Mortality data were subjected to chi-square analysis. A regression model fitting SAS procedure was employed to the data of RBC and albumin. Individual aquarium means were considered as the experimental unit.

## 3. Results

**Experiment 1 .**

Phytochemical screening tests revealed the presence of various active biocompounds in MFE including the tannins, flavonoids, alkaloids, saponins, volatile oils, phenolics, and terpenoids. The effect of the MFE supplement did not alter growth performance and heptosomatic index parameters (Table 2). Compared to the control diet, there was no effect (*P* > 0.05) on the body weight gains, feed intake, specific growth rate, feed:gain ratio, heptosomatic index, and total body length of the dietary MFE supplement at all levels of supplementation.

However, there was a significant effect of increasing dietary levels of MFE reducing the mortality rate. Treatments fed MFE-5 and MFE-7 diets had no mortality while control and MFE-2 showed 8.80% and 7.70%, respectively.

The effect of the dietary MFE treatments on the values of RBC, albumin, and total protein was significant (Table 3). There were linear effects (*P* < 0.05) of the dietary MFE treatments on the values of RBC, albumin, and total protein (*y* = 2.1 + 0.0045*x*;* R* ^2^ = 0.9747,* y* = 21.3 + 0.0017*x*;* R* ^2^ = 0.9582, and* y* = 40.1 + 0.0031*x*;* R* ^2^ = 0.9820, resp.). The value of Hg was higher (*P* < 0.05) for the treatment MFE-7 than for the other treatments (CNT, MFE-2, and MFE-5). There were no significant differences among the treatments in the values of WBC, Ht, globulin, and total lipids and in the activities of AST, ALT, and ALP.

**Experiment 2 .**

Body weight gain and FI depressed significantly (*P* < 0.05) when fish fed on CNT2-I, MFE-2-I, and MFE-5-I diets (Table 4). Feed conversion ratio impaired (*P* < 0.05) for the groups fed on CNT-2-I, MFE-2-I, and MFE-5-I diets. No significant difference (*P* > 0.05) was observed in HSI among dietary treatments. Total body length and SGR decreased (*P* < 0.05) in CNT-2-I, MFE-2-I, and MFE-5-I diets compared with the other groups.

Comparison of the three control treatments (CNT2, CNT2-I, and CNT2-I-A) revealed that the infection of fish with* A. hydrophila* (CNT2-I) increased (*P* < 0.05) the plasma values of WBC but decreased (*P* < 0.05) the values of all other measured parameters in plasma (RBC and Hb and Ht) and serum (total protein, albumin, and globulin) (Table 5). However, the addition of antibiotic to the* A. hydrophila* infected fish in the control treatment CNT2-I-A restored the values of all the measured parameters to the levels not different (*P* > 0.05) from the control treatment CNT2. Similarly, except for the RBC volume for the treatment MFE-5-I, all the measurements in the* A. hydrophila* infected fish in the MFE supplemented treatments with 5 or 7 g/kg DM (MFE-5-I and MFE-7-I) showed similar (*P* > 0.05) values compared with the control treatment CNT2-I-A. These values for the treatment MFE-7-I were also not different (*P* > 0.05) from those for the control treatment CNT2, but the value of WBC was significantly lower than control group (*P* < 0.05) and the values of other parameters (RBC, Hb and Ht, total protein, albumin, and globulin) were significantly higher (*P* < 0.05) for the treatment MFE-7-I than for the control treatment CNT2-I. The values of all the measured parameters were similar (*P* > 0.05) for the treatments MFE-2-I and CNT2-I.

**Figure 1:**
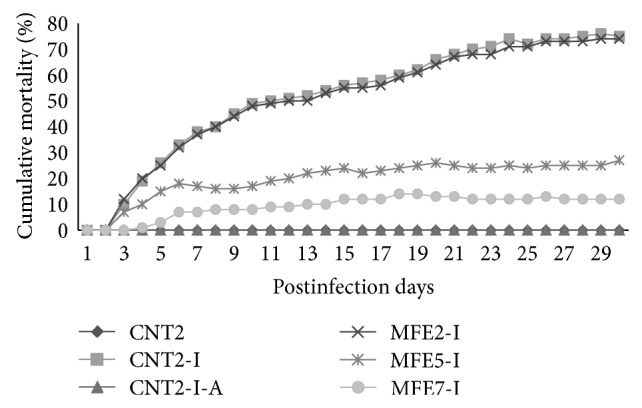
Experiment 2:percent cumulative mortality of fish during 30 days after infection with* Aeromonas hydrophila*. CNT2, control; CNT2-I, control with fish infected with* A. hydrophila* and fed the control diet without any additive; CNT2-I-A, control with fish infected with* A. hydrophila* and fed the control diet supplemented with the antibiotic oxytetracycline (1 g/199 g DM), while MFE-2-I, MFE-5-I, and MFE-7-I were control diet plus 2, 5, or 7 g/kg* Morus alba* foliage extract, respectively, and fish infected with* A. hydrophila*.

The cumulative mortality was high for the treatments CNT2-I and MFE-2-I (Figure 1), while that for the control treatment (CNT2) and CNT2-I-A was similar (no mortality). In comparison with the control treatment CNT2-I, the dietary supplementation of MFE at 5 g/kg DM decreased (*P* < 0.05) the cumulative mortality, but a higher decrease (*P* < 0.05) was obtained for the treatment MFE-7-I.

## 4. Discussion

The results of the present study clearly showed that the dietary supplements of the MFE enhanced survival rate and treatment of the African catfish. Furthermore, there were no adverse effects of this extract on the measured parameters. The results also showed that from the levels of the dietary inclusion of the MFE used in the present study (2, 5, and 7 g/kg DM) the dietary supplement of 7 g/kg DM was the most effective in improving the survival rate and in treating the infected fish with* A. hydrophila*. The inclusion of MFE in the diet failed to show any pronounced effect on the growth performance, though there were numerical improvements in the growth parameters of fish fed MFE supplemented diets. Several herbal plants also were tested for their growth promoting activity in aquatic animals such as Asian tiger shrimp (*Penaeus monodon*) by Citarasuet al. [43] and Jayaprakas and Euphrasia, [44] on Rohu (*Labeo rohita*) fish. In addition, Wu et al. [45] showed that* Gynostemma pentaphyllum*, a traditional herbal medicine in China, to grass carp feed resulted increased in FCR, weight gain, and growth rate.

Hematological assessment showed that, compared to control group, the values of RBC, Hg, albumin, and total protein were higher when the fish diet included MFE in the level of 7 g/kg DM; also, the values of RBC, albumin, and total protein were increased in fish receiving MFE at the dietary level of 5 g/kg DM. Goda [46] also showed that the dietary ginseng herb pronouncedly improved the hematological indices in tilapia. This reflects the improvements in the nutritional balance and in immunity enhancement in fish. Babatunde and Pond [47] reported that Hb and Ht values are correlated with the nutritional status of the animal and are directly related to the dietary nutritional balance. Equal values of WBC in all MFE treatments and the controls showed the ability of fish to the battle invasion of a disease without impairing phagocytosis due to the dietary supplementation with MFE.

Furthermore, increased activity of AST, ALT, and ALP could be due to damage of the liver, muscle [48], and other tissues [49]. In the present study, no pronounced changes were observed in total lipid, AST, ALT, and ALP due to the dietary supplements of MFE.

In fish, monitoring the indices such as hematological parameters can reveal general immune system condition. On the day 30 after the infection with* A. hydrophila* in Experiment 2, RBC, albumin, and total protein values were lower in the fish in the control treatment not treated with the antibiotic (CNT2-I) or in fish receiving the treatments MFE-2-I and MFE-5-I, than in fish receiving other treatments (CNT2, CNT2-I-A, and MFE-7-I). Ardó et al. [50] reported similar decreases in* Limanda ferruginea* fish challenged with various pathogens. Harikrishnan et al. [51] pointed out that infected fish fed low levels of a mixed herbal extract and untreated control fish showed pronounced reduction in the values of RBC and Hb and Ht, while the highest levels of the extract restored hematological and biochemical variables.

The RBC destruction leads to anemia [52]. However, using high levels of the dietary MFE (7 g/kg DM) in the present study improved this parameter to the point found in the control treatments CNT2 and CNT2-I-A. The white blood cells play a vital role in protection against all types of infection. In the present study, the counts of WBC in the control fish infected with* A. hydrophila *and untreated with the antibiotic (CNT2-I) or in fish in the treatment MFE-2-I increased compared to the uninfected control fish (CNT2), infected control fish treated with antibiotics (CNT-I-A), and fish in the treatments MFE-5-I and MFE-7-I. In addition, the infected fish treated with the antibiotic or the MFE at the dietary concentrations of 5 and 7 g/kg DM showed similar WBC counts as uninfected control fish (CNT2) or infected but the antibiotic treated control fish (CNT2-I-A). Similarly, Harikrishnan et al. [51] have shown such increase in WBC in the infected goldfish which restored to normal levels by the inclusion of herbal extract in the diet. This indicated the growth of pathogens stopped in the infected fish treated with the extract.

The percentage of cumulative mortality was lower in the control fish uninfected (CNT2), control fish infected with* A. hydrophila* and treated with antibiotic (CNT2-I-A), and infected fish receiving the MFE supplement at the levels of 5 and 7 g/kg DM (MFE-5-I and MFE-7-I) compared to other treatments (CNT2-I and MFE-2-I). Elsewhere, some studies pointed out that dietary Astragalus root, angelica root, and mixed herbal (*Curcuma longa*, Ocimumsanctum, and* Azadirachta indica*) extracts decreased mortality rate in Jian carp [53], large yellow croaker [54] and goldfish [51] after infection with a virulent strain of* A. hydrophila*.

Some* in vitro *studies conducted with MF revealed strong activity against some gram-negative and gram-positive bacteria [55, 56]. In addition, Kim et al. [57] found that the chloroform extracts of MF have strong antimicrobial activity against* Bacilllus subtilis*, while the extract with acetic acid is active against* Staphylococcus aureus* and* Escherichia coli. *The therapeutic properties of herbal extracts could be due to their phytochemical compounds [58, 59]. As shown the phytochemical screening of the presently used methanol extract of* Morus alba* detected the presence of tannins, flavonoids, alkaloids, saponins, volatile oils, phenolics, and terpenoids. Some of the bioactive compounds have been known for their antimicrobial activity. The tannins are strongly effective against bacteria [60], fungi [61], and viruses [62, 63]. Another* in vitro* study reported the antibacterial effects of the flavonoids on* Aeromonas spp*. such as* A. hydrophila* and* A. salmonicida* [64]. Furthermore, volatile oils and polyphenols can inhibit bacterial growth [65, 66].

In conclusion, the present results showed evidence that supplementation of MFE in the diet of African catfish is safe, improves survival rate, and cures the fish after infection with motile aeromonad septicaemia caused by* A. hydrophila*. Consequently, MFE at the level of 7 g/kg DM might be a potentially valuable dietary supplement to cure the infected fish with* A. hydrophila*.

## Figures and Tables

**Table 1:** Ingredient composition (g kg^−1^) and proximate composition of control diet (dry matter basis).

Ingredients	Concentrations
Corn grain^3^	320
Soybean meal^4^	380
Fish meal^5^	170
Blood meal^6^	50
Palm oil^7^	50
Mineral premix^1^	10
Vitamin premix^2^	10
Binder	10
Chemical analysis (%)	
Dry matter	88.1
Crude protein	35.0
Crude lipid	7.67
Crude fiber	6.71
Ash content	7.10
Digestible energy (kcal kg^−1^)	3498

^1^Contains per kg of premix: dibasic calcium phosphate, 500 g; calcium carbonate, 215 g; sodium chloride, 40 g; potassium chloride, 90 g; magnesium hydroxide, 124 g; iron sulfate, 20 g; zinc sulfate, 4 g; manganese sulfate, 3 g; cobalt sulfate, 0.02 g; potassium iodide, 0.04 g; sodium selenite, 0.03 g; sodium fluoride, 1 g.

^2^Contains per kg premix: retinol palmitate, 2,500,000 IU; cholecalciferol, 500,000 IU; tocopherol acetate, 30 g; menadione, 2 g; thiamine, 2 g; riboflavin, 5 g; panthotenic acid, 10 g; niacin, 5 g; pyridoxine, 4 g; folic acid, 2 g; cyanocobalamin, 4 mg; ascorbic acid, 20 g; biotin, 200 mg; inositol, 80 g.

^1,2,3,4,5^were purchased from Sigma (MO, USA).

^6^were purchased from Cameron Agric Complex Inc.

^7^Malaysian palm oil.

**Table 2:** Experiment 1: growth performance and hepatosomatic index in African catfish fed diets with different amounts (2, 5, or 7 g/kg dry matter) of *Morus alba* foliage extract (MFE).

Parameters	Diets^1^			
	Control	MFE-2	MFE-5	MFE-7
Body weight gain (g)	80.5 ± 5.2	80.0 ± 3.7	85.3 ± 5.5	83.1 ± 4.0
Feed intake (g 60/days)	97.9 ± 4.2	96.1 ± 2.4	99.7 ± 3.9	98. 2 ± 5.1
Feed conversion ratio	1.22 ± 0.05	1.20 ± 0.09	1.17 ± 0.04	1.18 ± 0.08
Specific growth rate (%)	3.93 ± 0.32	3.97 ± 0.18	4.01 ± 0.21	3.95 ± 0.16
Mortality (%)	8.80 ± 1.03^a^	7.70 ± 1.51^a^	0.00^b^	0.00^b^
Hepatosomatic index (%)	1.65 ± 0.08	1.56 ± 0.05	1.59 ± 0.07	1.67 ± 0.09
Total body length (cm)	18.6 ± 1.7	18.0 ± 1.5	19.9 ± 1.8	19.5 ± 2.3

^a,b^Means within the same row with different superscript letters are significantly different (*P* < 0.05).

^1^MFE-2, MFE-5, and MFE-7 were control diet plus 2, 5, or 7 g kg^−1^ dry matter of *Morus alba* foliage extract, respectively.

Values are the treatment means ± standard error.

**Table 3:** Experiment 1: hematological and biochemical indices in African catfish fed different amounts (2, 5, or 7 g/kg dry matter) of *Morus alba* foliage extract (MFE).

Parameters	Diets^1^			
	Control	MFE-2	MFE-5	MFE-7
Red blood cells (10^6^/*μ*L)	2.16 ± 0.06^b^	2.31 ± 0.07^b^	2.44 ± 0.09^ab^	2.58 ± 0.08^a^
White blood cells (10^5^/*μ*L)	3.62 ± 0.10	3.51 ± 0.08	3.59 ± 0.05	3.55 ± 0.06
Hemoglobin (g/dL)	8.26 ± 0.5^b^	8.29 ± 0.9^b^	8.12 ± 0.6^b^	9.78 ± 0.8^a^
Hematocrit (%)	28.1 ± 5.3	31.3 ± 6.5	36.9 ± 4.6	38.7 ± 5.1
Albumin (g/100 mL)	17.4 ± 1.8^b^	18.5 ± 1.4^b^	22.5 ± 2.1^a^	26.1 ± 2.3^a^
Globulin (g/100 mL)	20.7 ± 3.1	17.1 ± 3.4	21.0 ± 3.0	19.6 ± 2.2
Total protein (g/100 mL)	38.1 ± 3.6^b^	35.6 ± 3.5^b^	43.5 ± 2.0^ab^	45.7 ± 2.6^a^
Total lipids (g/L)	0.87 ± 0.05	0.90 ± 0.06	0.93 ± 0.08	0.81 ± 0.09
^ 2^AST (IU/L)	17.0 ± 8.2	22.9 ± 7.3	31.4 ± 8.5	29.2 ± 6.7
^ 3^ALT (IU/L)	20.6 ± 4.2	18.5 ± 6.5	23.7 ± 3.9	26.1 ± 5.0
^ 4^ALP (IU/L)	30.9 ± 2.7	33.6 ± 3.9	28.8 ± 2.8	32.2 ± 2.4

^a,b^Means within the same row with different superscript letters are significantly different (*P* < 0.05).

^1^MFE-2, MFE-5, and MFE-7 were control diet plus 2, 5, or 7 g/kg of dry matter of *Morus alba* foliage extract, respectively.

Values are the treatment means ± standard error.

^2^AST: aspartate aminotransferase; ^3^ALT: alanine aminotransferase; ^4^ALP: alkaline phosphatase.

**Table 4:** Experiment 2: effects of experimental diets on growth response and hepatosomatic index in African catfish fed diets with different amounts (2, 5, and 7 g/kg of dry diet) of *Morus alba* extract (MFE) on day 30 after infection with *A. hydrophila*.

Parameters	Diets^1^					
	CNT2	CNT2-I	CNT2-I-A	MFE-2-I	MFE-5-I	MFE-7-I
Body weight gain (g)	45.6 ± 2.2^a^	36.9 ± 1.8^b^	48.4 ± 1.6^a^	38.1 ± 3.0^b^	40.0 ± 2.7^b^	46.9 ± 2.5^a^
Feed intake (g/30 days)	57.1 ± 2.4^a^	49.5 ± 2.1^b^	59.2 ± 3.3^a^	50.9 ± 2.8^b^	52.0 ± 2.6^b^	58.6 ± 1.8^a^
Feed conversion ratio	1.25 ± 0.04^b^	1.34 ± 0.02^a^	1.22 ± 0.03^b^	1.33 ± 0.05^a^	1.30 ± 0.04^ab^	1.25 ± 0.02^b^
Specific growth rate (%)	6.20 ± 0.09^a^	5.70 ± 0.13^b^	6.30 ± 0.15^a^	5.63 ± 0.19^b^	5.90 ± 0.17^b^	6.23 ± 0.11^a^
Hepatosomatic index (%)	1.65 ± 0.10	1.60 ± 0.07	1.68 ± 0.14	1.57 ± 0.09	1.60 ± 0.12	1.66 ± 0.14
Total body length (cm)	15.9 ± 1.0^a^	13.2 ± 0.8^b^	16.8 ± 0.7^a^	13.0 ± 0.6^b^	13.5 ± 0.9^b^	16.4 ± 0.5^a^

^a,b^Means within the same row with different superscript letters are significantly different (*P* < 0.05).

Values are the mean ± standard error.

CNT2, control; CNT2-I, control with fish infected with *A.hydrophila* and fed the control diet without any additive; CNT2-I-A, control with fish infected with *A. hydrophila* and fed the control diet supplemented with the antibiotic oxytetracycline (1 g/199 g DM), while MFE-2-I, MFE-5-I, and MFE-7-I were control diet plus 2, 5, or 7 g/kg *Morus alba* foliage extract, respectively, and fish infected with *A. hydrophila*.

**Table 5:** Experiment 2: hematological and biochemical indices in African catfish fed different amounts (2, 5 or 7 g/kg dry matter) of *Morus alba* foliage extract (MFE) with infection of fish with *A. hydrophila* (I) and compared to controls without or with a supplement of antibiotic oxytetracycline. (A) and without or with infection of fish with *A. hydrophila* (I).

Parameters	Diets^1^					
	CNT2	CNT2-I	CNT2-I-A	MFE-2-I	MFE-5-I	MFE-7-I
Red blood cells (10^6^/*μ*L)	2.87 ± 0.06^a^	2.45 ± 0.04^b^	2.83 ± 0.03^a^	2.52 ± 0.07^b^	2.49 ± 0.05^b^	2.79 ± 0.03^a^
White blood cells (10^5^/*μ*L)	3.62 ± 0.07^b^	4.05 ± 0.08^a^	3.50 ± 0.12^b^	3.98 ± 0.09^a^	3.88 ± 0.11^a^	3.54 ± 0.10^b^
Hemoglobin (g/dL)	10.04 ± 0.65^a^	8.65 ± 0.47^b^	9.96 ± 0.30^a^	8.45 ± 0.62^b^	9.52 ± 0.51^ab^	10.21 ± 0.75^a^
Hematocrit (%)	43.2 ± 3.4^a^	30.7 ± 3.9^b^	45.0 ± 2.6^a^	27.6 ± 3.3^b^	37.3 ± 3.5^ab^	38.7 ± 4.8^a^
Total protein (g/100 mL)	46.6 ± 2.9^ab^	32.2 ± 3.0^c^	52.2 ± 2.4^a^	31.0 ± 2.8^c^	35.5 ± 2.5^b^	48.3 ± 3.1^a^
Albumin (g/100 mL)	22.7 ± 2.6^a^	14.8 ± 3.0^b^	25.9 ± 2.7^a^	15.2 ± 2.2^b^	17.2 ± 2.4^b^	23.1 ± 3.2^a^
Globulin (g/100 mL)	23.9 ± 3.2^a^	17.4 ± 3.1^b^	26.3 ± 2.1^a^	15.8 ± 3.4^b^	18.3 ± 2.6^ab^	25.2 ± 3.0^a^

^a,b,c^Means within the same row with different superscript letters are significantly different (*P* < 0.05).

^1^CNT2, control in Experiment 2; CNT2-I, fish infected with *A. hydrophila* and fed the control diet without any additive; CNT2-I-A, fish infected with *A. hydrophila* and fed the control diet supplemented with the antibiotic oxytetracycline (1 g/199 g DM), while MFE-2-I, MFE-5-I, and MFE-7-I were control diet plus 2, 5, or 7 g/kg *Morus alba* foliage extract, respectively, and infected with *A.hydrophila*. Values are the treatment means ± standard error.
